# Transmission from centenarians to their offspring of mtDNA heteroplasmy revealed by ultra-deep sequencing

**DOI:** 10.18632/aging.100661

**Published:** 2014-05-13

**Authors:** Cristina Giuliani, Chiara Barbieri, Mingkun Li, Laura Bucci, Daniela Monti, Giuseppe Passarino, Donata Luiselli, Claudio Franceschi, Mark Stoneking, Paolo Garagnani

**Affiliations:** ^1^ Department of Biological, Geological and Environmental Sciences, Laboratory of Molecular Anthropology and Centre for Genome Biology, University of Bologna, Bologna, Italy; ^2^ Department of Evolutionary Genetics, Max Planck Institute for Evolutionary Anthropology, Leipzig, Germany; ^3^ Department of Experimental, Diagnostic and Specialty Medicine (DIMES), University of Bologna, Bologna, Italy; ^4^ Interdepartmental Centre “L. Galvani” for Integrated Studies of Bioinformatics, Biophysics and Biocomplexity (CIG), University of Bologna, Bologna, Italy; ^5^ Department of Experimental and Clinical Biomedical Sciences, University of Florence, Florence, Italy; ^6^ Department of Cell Biology, University of Calabria, Rende, Italy; ^7^ IRCCS, Institute of Neurological Sciences of Bologna, Bologna, Italy; ^8^ CNR, Institute of Organic Synthesis and Photoreactivity (ISOF), Bologna, Italy; ^9^ IGM-CNR Institute of Molecular Genetics, Unit of Bologna IOR, Bologna, Italy; ^10^ Center for Applied Biomedical Research (CRBA), St. Orsola-Malpighi University Hospital, Bologna, Italy

**Keywords:** mtDNA heteroplasmy, longevity, aging, transmission, centenarians

## Abstract

The role that mtDNA heteroplasmy plays in healthy aging, familial longevity and the heritability patterns of low levels heteroplasmy in the elderly are largely unknown. We analyzed the low levels of mtDNA heteroplasmy in blood in a cohort of centenarians, their offspring and a group of offspring of non long-lived parents, characterized by a less favorable health phenotype. The aims of this study are to: (i) investigate the transmission of low level heteroplasmies in the elderly; (ii) explore the association of heteroplasmy with age and longevity and (iii) investigate heteroplasmy patterns in these three groups. We sequenced a 853 bp mtDNA fragment in 88 individuals to an average coverage of 49334-fold, using quality control filtering and triplicate PCR analysis to reduce any methodological bias, and we detected 119 heteroplasmic positions with a minor allele frequency ≥ 0.2%. The results indicate that low-level heteroplasmies are transmitted and maintained within families until extreme age. We did not find any heteroplasmic variant associated with longevity and healthy aging but we identified an unique heteroplasmy profile for each family, based on total level and positions. This familial profile suggests that heteroplasmy may contribute to familial longevity.

## INTRODUCTION

Many studies have demonstrated that human healthy ageing and longevity are familial traits inherited across generations [1-4] and that centenarian offspring have higher chances for healthy aging[5, 6]. To assess the factors that can contribute to this phenotype, many studies of long-lived families have been carried out from several perspectives, such as genomics [7], metabolomics [8], epigenetics [9], gut microbiome [8, 10] and mitochondrial DNA (mtDNA) variability [11]. Among these domains it is well established that the physiological decline of mitochondrial machinery plays a critical role in the aging process, given its role in providing energy to cells and tissues and the high production of reactive oxidative species. One factor that can contribute to age-related mitochondrial dysfunction is the accumulation of mtDNA mutations linked to errors in the replication machinery [12-14]. MtDNA heteroplasmy, *i.e.* intra-individual variation in mtDNA sequences, may be of relevance.

Some studies have addressed the role that mtDNA heteroplasmy may play in promoting longevity [15-18]. A possible beneficial effect on healthy aging is that mtDNA heteroplasmy represents a reservoir of genetic variability, that can bring new functions and increase the capacity of cells to cope with environmental and physiological stressors during life [15, 17, 19, 20] but the forces that drive the fate of heteroplasmic mutations are still a matter of debate [21, 22]. Moreover recent studies demonstrated that the heteroplasmy pattern is inherited [23-26], including low-frequency heteroplasmic variants [27]. This new insight identifies heteroplasmy as a possible genetic contributor to familial longevity, but to date there are no data that show that the inherited heteroplasmic profile is conserved until extreme age. Various forces contribute to heteroplasmy, but the extent to which heteroplasmies are inherited or arise via new mutations is still uncertain, making the study of heteroplasmy in the extreme elderly even more pertinent. The heteroplasmy pattern observed in middle-age individuals is a mixture of both inherited and somatic mtDNA mutations that can potentially change according to tissue composition and cell clonality [28]. It is because of the bottleneck that occurs during postnatal folliculogenesis [29] and the somatic events that naturally occur during life [20, 30] that the frequency of heteroplasmies can change from one generation to another [20].

A further challenge in the study of heteroplasmy and aging is represented by technological limits in heteroplasmy detection. Previous technologies (such as DHPLC or pyrosequencing) did not allow a high site-specific resolution of very low-level heteroplasmic variants [31]. Thus, the advent of next-generation sequencing technologies, with the potential for very high coverage, seems ideal for investigating heteroplasmic events in very old people [27].

In the present study we analyzed 30 female centenarians (CENT), 30 offspring of these centenarians (CO) and a control group of 28 offspring of non-long-lived parents (ONLL), *i.e*. offspring of parents who died before reaching the average life expectancies for their cohort. We detected heteroplasmic positions by an ultra-deep re-sequencing method on the Illumina GA platform, focusing on 853 bp of the mtDNA control region (including HV1 and part of HV2). We focused on this region because heteroplasmies occur preferentially at positions with high mutation rates, in particular in the mtDNA control region, and because this region was already analyzed in previous studies of longevity [17, 19, 32].

The aims of this study are three-fold: (i) to address differences and similarities between centenarians (whose extended lifespan could be the result of a better adaptation to environmental stress and stimuli [33]), their offspring, characterized by a better health phenotype [34] and the offspring of non-long-lived parents; (ii) to describe mtDNA heteroplasmy patterns in extreme longevity; and (iii) to address if low levels of mtDNA heteroplasmy are trasmitted, maintained and detectable in very old mother offspring pairs (centenarians and their offspring) and how this mechanism could contribute to human longevity.

## RESULTS

### Reliability of mtDNA heteroplasmies detected by ultra-deep resequencing

Many studies raise several doubts related to ultra-deep sequencing based on PCR amplicons [31, 35] such as a biased amplification of nuclear mitochondrial pseudogenes (numts), or a very high number of false positives linked to a high sequence error rate or biased PCR amplification. To reduce the possibility of amplifying numts we first tested the pair of primers by in silico PCR to exclude non-specific amplifications, then the PCR products were checked on agarose gels to exclude the presence of non-specific amplicons. Rigorous criteria were used to distinguish true heteroplasmies from sequencing error, such as double strand validation and filtering (Methods), previously implemented in other studies [36]. Most importantly, to evaluate the potential for PCR errors and to obtain an estimate of the number of false positives, 3 different PCR products from 12 samples were sequenced and the concordance of heteroplasmic sites (identified using 0.2% as the lowest heteroplasmy threshold [24, 27]) between replicates checked.

Thus sensitivity and specificity of heteroplasmy detection were calculated. Sensitivity (*i.e*. a measure of the proportion of heteroplasmic sites correctly identified as such) provides the false negative rate while specificity (*i.e*., a measure of the proportion of homoplasmic sites correctly identified as such) provides the false positive rate. Because there is no suitable alternative method to detect very low levels of heteroplasmy (*i.e*. 0.2%), in the calculation of sensitivity and specificity levels a site was defined as “true heteroplasmic” if heteroplasmy was supported by at least 2 of the 3 observations. Mean sensitivity and specificity values were calculated as 0.95 ± 0.09 and 1.00 ± 0.0025 respectively. These high values obtained here support the possibility to detect and quantify heteroplasmic point mutations with high accuracy (for detailed analysis see Table 1S).

### Heteroplasmy profiling in centenarians, centenarian offspring and offspring of not long-lived parents

**Figure 1:**
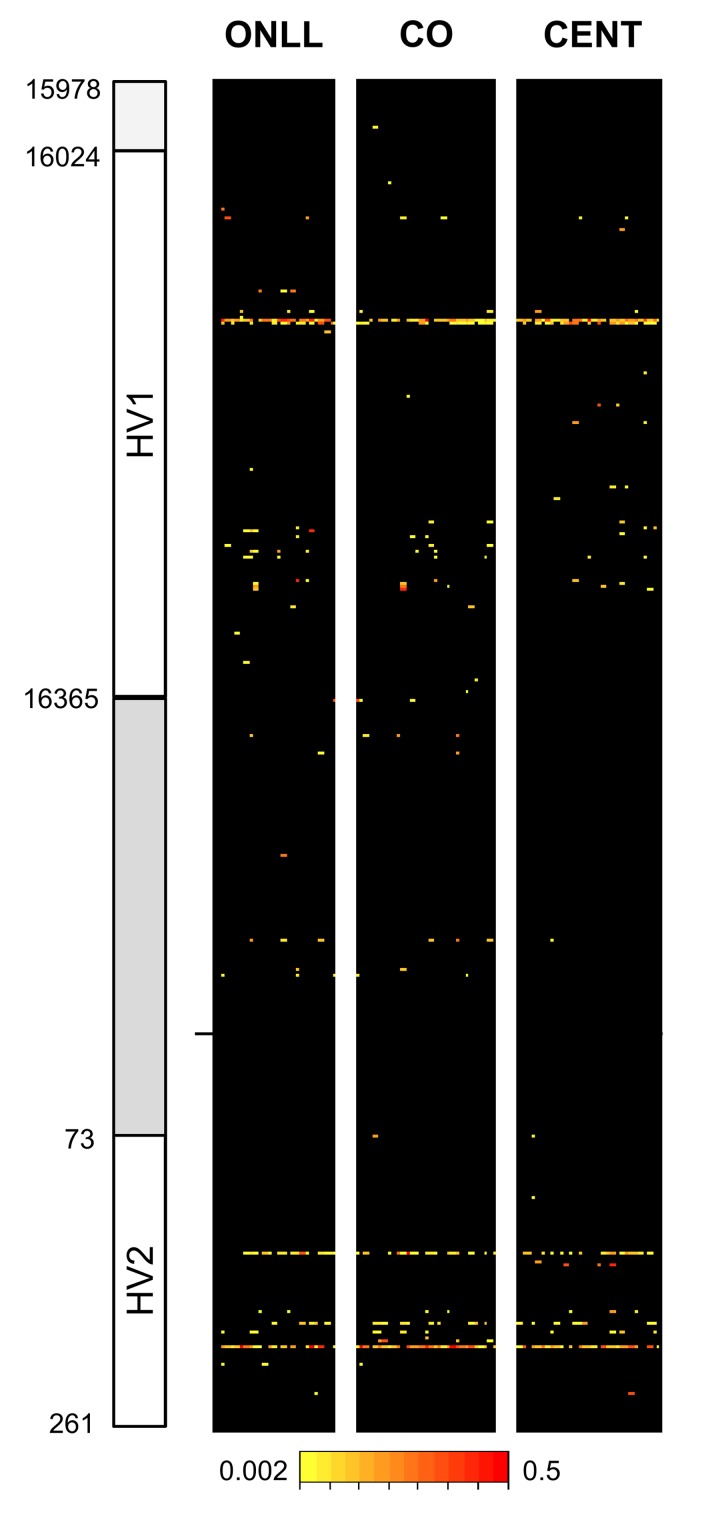
Overview of heteroplasmy pattern in the 853 bp region considered Heteroplasmic positions are indicated with different colors according to the level of heteroplasmy. Positions are reported on the y-axis while individuals are reported on the x axis.

After quality control (see Methods) there were results for 30 CENT, 30 CO and 28 ONLL. Heteroplasmy was defined with a minimum threshold of 0.2%, resulting in 119 heteroplasmic positions in the 853 bp fragment analyzed (Figure 1). In particular, we identified 75 heteroplasmic sites in CENT, 101 in CO and 72 in ONLL. We then performed a Fisher-exact test to assess if HV1 (positions: form 15978 to 16364), the intermediate region (positions: from 16365 to 72) and HV2 (positions: from 73 to 261) have similar proportions of heteroplasmic sites. The test showed that HV1 and HV2 are enriched in heteroplasmic sites (OR = 1.25; CI 95% 1.10-1.43 and OR = 1.66; CI 95% 1.43-1.92 respectively) when compared to the intermediate region (OR = 0.24 CI 95% 0.18-0.32). Unsupervised hierarchical clustering was also performed to evaluate the distance between individuals (considering minor allele frequency); CENT, CO and ONLL individuals did not cluster according to group (Figure 1S).

Since heteroplasmies occur preferentially at positions with high mutation rate [31, 32] we explored the correlation between the incidence of heteroplasmy and mutation rates (MR) in our cohort. Mean MR was first calculated considering all positions sequenced in this study (853 bp) and then considering all heteroplasmic sites (Table 1). Similarly we calculated the average MR in HV1, HV2 and the intermediate region considering all sites and then selecting only the heteroplasmic positions for each region.

**Table 1:** Average mutation rate calculated in all sites and only in heteroplasmic sites considering the entire region sequenced, HV1, HV2, and the intermediate region (from position 16365 to position 73).

	Average Mutation Rate	
	All Sites	Heteroplasmic sites
Entire region (853bp)	3.6	15.9 *
**HV1**	4.7	14.2 *
**HV2**	6.2	30.9 *
**Intermediate region**	1.6	6.4

* **p < 0.0001 (Mann Whitney test)**

**Figure 2:**
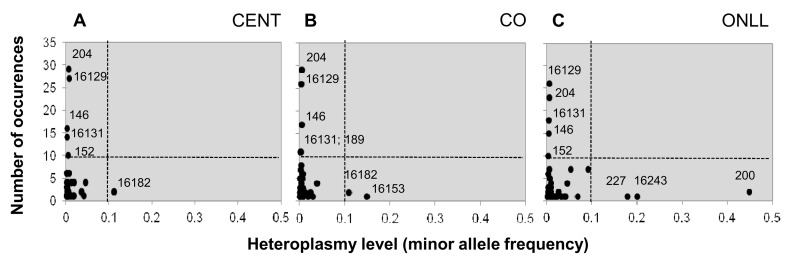
Heteroplasmic positions in the 3 groups Heteroplasmic positions plotted according to mean heteroplasmy level and number of occurrences in CENT (**A**), CO (**B**) and ONLL (**C**) respectively.

In order to combine information on both the incidence and the level of heteroplasmy, each position was plotted according to the number of heteroplasmic events and the mean heteroplasmy level in each group (Figure 2). The plot consists of four regions: the upper left quarter shows positions that occur frequently and are characterized by a low level of heteroplasmy; the upper right quarter is empty, indicating that there are no positions characterized by both a high frequency and a high level of heteroplasmy; the lower right quarter includes positions with high heteroplasmy levels but that occur in only a few individuals; and the lower left quarter includes most of the positions, which occur in only a few individuals and with a low heteroplasmy level.

Pearson's correlation between the number of occurrences in CENT, CO and ONLL and the MR of each position was calculated (r CENT = 0.43, p value CENT = 1.012*10^−6^; r CO = 0.46, p value CO = 1.51*10^−7^; r ONLL = 0.47, p value ONLL = 5.442*10^−8^ respectively).

We then compared heteroplasmy levels in CO and ONLL and ANOVA was performed to determine if the level of heteroplasmy at each position differs between the two groups. Only position 16131 showed a weak significant nominal p values (p value = 0.0062) that is not significant when corrected for multiple test comparisons (Table 2).

**Table 2:** Significant results of the ANOVA considering the minor allele frequency site by site.

mtDNA position	p value	Significant comparisons
**153**	0.0299	CENT-ONLL
**204**	0.047	CENT-ONLL
**16129**	0.028	CENT-CO
**16131**	0.025	CO-ONLL

We next compared each of the offspring groups (CO and ONLL) to centenarians (CENT) to assess whether heteroplasmy levels increase with age. Only positions 153, 204 and 16129 showed weak significant nominal p values (< 0.05 in the ANOVA test) that are not significant when we corrected for multiple test comparisons (Table 2).

To determine if heteroplasmy increases with age we compared the total number of heteroplasmic sites across the three groups by ANOVA; no significant differences among groups were detected (p value = 0.798). We tested for correlations between the age of all individuals (irrespective of group) and the heteroplasmy level for each site. Two positions showed a moderate trend with age: 204 (r = 0.20, p value = 0.06) and 16129 (r = 0.30, p value = 0.0054) (Figure 2S). In addition, the correlation between age and the number of heteroplasmic sites for each individual was not significant (r = 0.17, p value = 0.11).

### Heteroplasmy in centenarians and their offspring

#### Total heteroplasmy analysis

**Figure 3:**
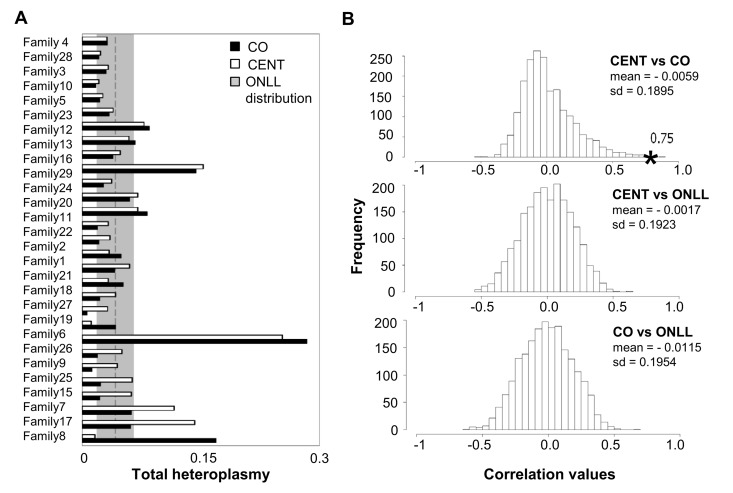
Total heteroplasmy in centenarians and their offspring and reshuffling analysis (**A**) Total heteroplasmy in centenarians and their offspring. White bars: centenarians; black bars: matched offspring. The dashed line and shading show the mean level and standard deviation of total heteroplasmy in ONLL. (**B**) Frequency distribution of correlation values based on 2000 reshufflings of total heteroplasmy values for CENT and unrelated CO, CENT and ONLL and CO and ONLL. Mean and standard deviation of the distribution is reported. The black star indicates the observed correlation value matching each centenarian with her own offspring.

One centenarian and one centenarian's offspring could not be considered in this analysis (for more details see Methods), therefore in the following analyses we considered 29 mother-offspring pairs. Total heteroplasmy was then calculated for each individual (total heteroplasmy = sum of minor allele frequencies at all heteroplasmic sites) to quantify the mutation load of the entire mtDNA genome. These total heteroplasmy values are significantly correlated between centenarians and their offspring (Figure 3A; r = 0.75, p = 2.88*10^−6^). To further validate this result, the CENT and CO individuals were reshuffled 2000 times, matching centenarians with unrelated offspring, and the correlation values were calculated. This analysis (Figure 3B) indicates that the observed correlation between CENT and their true offspring in total heteroplasmy is indeed unlikely to arise by chance. In addition, correlation values of 2000 possible combinations of CENT-ONLL and CO-ONLL are centered around zero (Figure 3B), consistent with an absence of any correlation in these comparisons. This analysis suggests that the total heteroplasmy values are maintained in families.

#### Number of heteroplasmic sites

**Figure 4:**
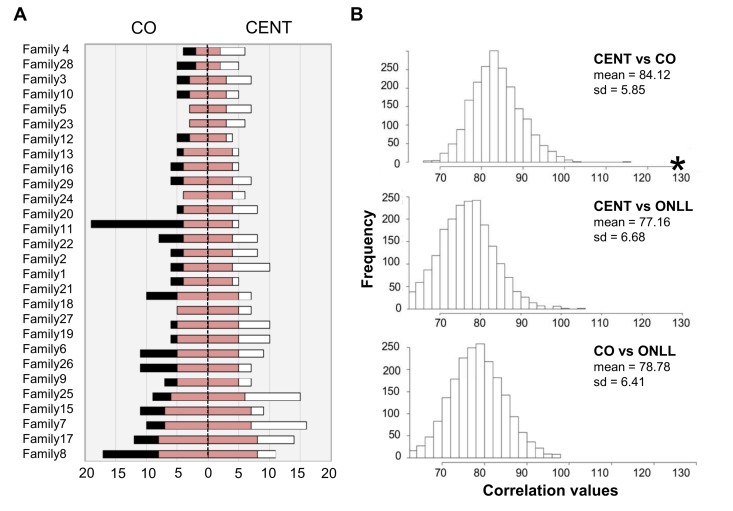
The number of common heteroplasmic sites shared by CENT and CO and frequency distributions of TNCS in all the reshuffled pairs (**A**) The white bar indicates the number of heteroplasmic position in CENT, the black bar indicates the number of heteroplasmic sites in CO. CENT and CO are here matched according to consanguinity (x-axis). The red bar shows the number of shared heteroplasmic sites in each parent-offspring pair. (**B**) Frequency distributions of TNCS in all the reshuffled pairs considering CENT and unrelated CO, CENT and ONLL, and CO and ONLL. Black star = total number of common heteroplasmic sites matching centenarians with their own offspring.

Centenarians and their offspring were then analyzed to assess shared heteroplasmic positions. The total number of positions shared by CENT and CO over 29 families is 130 (total number of common sites, TNCS); the results for each family are depicted schematically in Figure 4A, and a detailed list is provided in Table 2S. The TNCS was recalculated 2000 times, matching randomly subjects of each the two groups (Figure 4B). This analysis indicates that more TNCS are shared by centenarians and their offspring than between any other combinations of individuals.

#### Heteroplasmy analysis site by site

**Figure 5:**
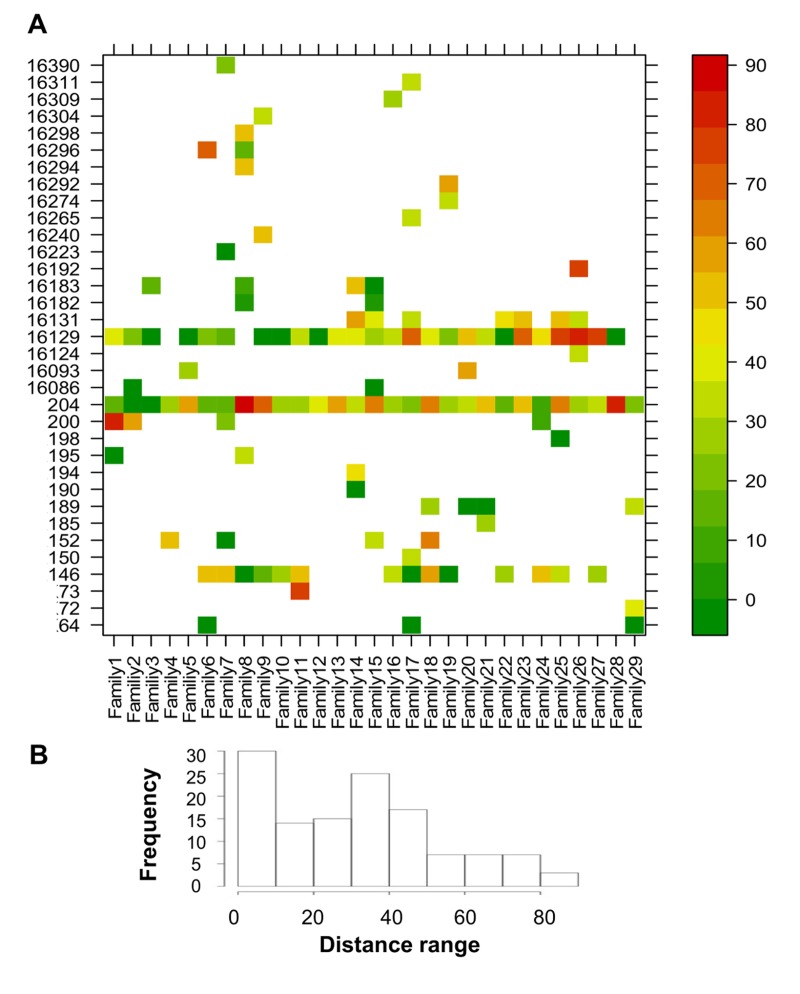
CENT and CO distances of positions that showed heteroplasmy in both individuals Distance values were calculated as the ratio between the absolute difference between CENT and CO MAF and the higher MAF value of the two (expressed as percentage). (**A**) Heteroplasmy distances plotted for each position and colored according to the scale bar on the right. (**B**) Frequency distribution of the distance values.

To evaluate the similarity in minor allele frequencies (MAF) across sites between each CENT mother and her offspring, a site-by-site distance was calculated (Figure 3S). The ratio between the absolute difference in MAF (abs[CENT-CO]) and the highest MAF of the two was calculated as a measure of the “distance” between each CENT and CO (Figure 5A). Each pair shows *private sites* that are heteroplasmic in CENT and CO with variable values of concordance but different between pairs. Some positions, such as 146, 204 and 16129, are heteroplasmic in most pairs (*public sites*). The frequency distribution of all distance values is reported in Figure 5B; only positions that show heteroplasmy in both individuals were considered in this analysis.

## DISCUSSION

To date, several studies have showed that hetero-plasmies tend to accumulate during human aging in many tissues, such as brain, heart, skeletal muscles and blood [20, 27, 37-41], but there is still controversy regarding the cause of such age related accumulation of heteroplasmic mutations. The most recent hypothesis suggests that replication errors arise during life and accumulate by clonal expansion and genetic drift, placing the accumulation of ROS damage in the background [14, 42]. This scenario becomes even more intricate when we consider that even low-level heteroplasmies can be inherited [27]. Taking into account the high number of independent mtDNA copies, it is worthwhile to sum up the possible sources of mtDNA variability: (i) heteroplasmy derives from inherited variants and *de novo* mutations [20]; (ii) this variability originates prenatally, from the very first stages of cell divisions and it is modified subsequently during life by segregating events; and (iii) mother and offspring share similars pattern of somatic mtDNA variability.

Given these assumptions we explored whether the inherited heteroplasmy profile is preserved in the elderly or if *de novo* mutations overcome the inherited variants. Moreover we assessed the correlation between heteroplasmy and longevity, and we focused on the identification of specific heteroplasmic profiles for centenarians, their offspring and offspring of non-long lived parents to highlight features linked to familial predisposition to longevity [9, 39, 43, 44]. Centenarian offspring have higher chances for healthy aging [5, 6, 45] even when they share a similar lifestyle with the general population [46]. Moreover clinical [34] and molecular data [8-10] demonstrated that they are characterized by a better health status than offspring of non long lived parents. Applying next-generation sequencing to this cohort, we investigated human mtDNA heteroplasmy transmission and association with longevity. We obtained extremely high coverage (average 49334 fold per position) and replicates analysis demonstrates the possibility to detect very low levels of heteroplasmy (down to 0.2%).

The main results of this paper indicate that low-level heteroplasmies in blood are detectable, transmitted and maintained within families until extreme age. Moreover we did not find any heteroplasmic variant associated with longevity and healthy aging but we identified an unique heteroplasmy profile for each family, resulting from a combination of total heteroplasmy level and specific heteroplasmic positions.

Regarding the technological approach in the first analysis we considered triplicate samples to demonstrate the accuracy and reliability of the method used. We performed double strand validation that considerably reduces the impact of sequencing error [36] and then we used replicates to assess the level of sensitivity and specificity. Sequencing errors, assembly strategies, biased PCR amplification and stochastic effects are unlikely to produce false positives under our criteria for detecting heteroplasmy. The resulting data show that a small fraction of heteroplasmic sites could not be reproduced (possibly because they are false negative), but sites called as *heteroplasmic* are likely to be real mutations. Our results confirmed the possibility to detect with high accuracy a low level of mutation (< 2% of minor allele frequency), as also shown in a recent study [27]. It is worthwhile to note that previous studies, with relatively high coverage, did not show the error rate, sensitivity and specificity and it is likely that these values are directly dependent on the mean coverage. From a technical point of view we can conclude that sequencing depth is very important to identify and to quantify low-level heteroplasmic variants with high accuracy.

We analyzed heteroplasmies focusing on the non-coding region (from position 15978 to position 261). We observed that HV1 and HV2 are enriched in heteroplasmic sites when compared to the intermediate region. We also observed that in our cohort heteroplasmies tend to occur in positions characterized by a high mutation rate. This phenomenon was described previously [31, 32] suggesting that mutation is the major driving force behind heteroplasmies and that subsequent drift to high frequencies in an individual can lead to polymorphisms among individuals [31].

We then analyzed heteroplasmy patterns in our groups (CENT, CO and ONLL). Comparing the minor allele frequency between groups via ANOVA, the obtained *p values* were not significant, (when corrected for multiple test comparisons), indicating the overall absence of significant heteroplasmy differences between groups. We did not find any differences also in the number of heteroplasmic sites between groups. We also analyzed the heteroplasmic variation across ages and we find only two sites that showed a modest correlation with age, positions 204 and 16129. A previous study that measured heteroplasmies in blood of individuals with different ages (0-60 yrs) found several heteroplasmic sites whose frequency correlates with age [20]. However even if we did not observe a clear pattern of age-related heteroplasmy accumulation, we cannot exclude that heteroplasmies accumulate during life because of the limited age range here considered (average age = 80.7 ± 15.1).

**Figure 6:**
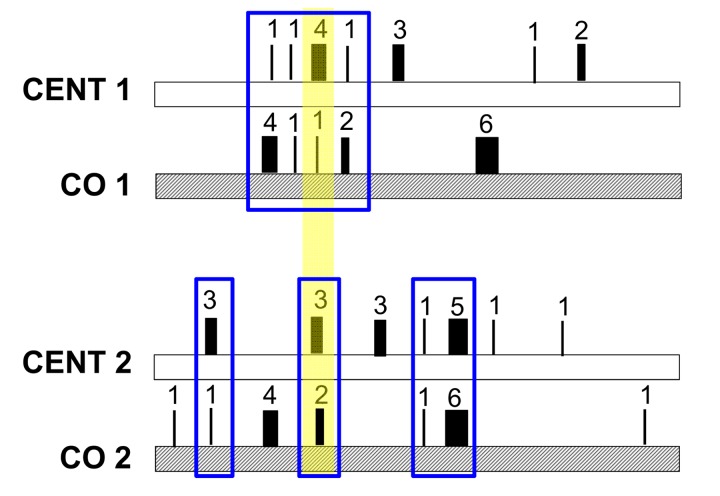
Model of the mechanism of heteroplasmic patterns in blood of long-lived families The number and thickness of each rectangle represents the different heteroplasmic level of each site, while the sum of all numbers represents the total heteroplasmy value. Total heteroplasmy is more similar within parents-offspring pairs than between pairs (CENT1 = 13 and CO1 = 14 while CENT2 = 17 and CO2 = 16). Each family shares a common core of heteroplasmic sites (here marked in blue). This core is composed of two types of heteroplasmic sites (i) *public heteroplasmic positions* that all pairs share (here marked with a yellow shadow) and (ii) *private heteroplasmic sites* that characterize each family.

Regarding the heteroplasmy pattern in families (mother-offspring pairs), we found a clear signature of heteroplasmy inheritance in consideration of the centenarians-offspring concordance of both total heteroplasmies levels and of the mutated loci (heteroplasmy profile). This concordance was tested and confirmed by reshuffling analyses. Our data suggest that families share a *common core* of heteroplasmic sites that could be divided, according to their frequency in the cohort, into (i) *public heteroplasmic positions* that all parent-offspring couples shared (such as positions 146, 204 and 16129) and (ii) *private heteroplasmic sites* that are common to only one or few families. Figure 6 simplifies the heteroplasmic profiles we observed. We note that public heteroplasmic positions may reflect recurrent mutations as well as inherited hetreroplasmies [47]. These data are consistent with previous studies [17, 19] that found that a high concordance of heteroplasmy can be observed within families whose ages are shifted toward the last decades of life. However, the use of DHPLC (Denaturating High Performance Liquid Chromatography) in these previous studies did not allow the precise identification of heteroplasmic positions, as we did in the present study. Taken together the groups comparison and the familial analysis suggested that the heteroplasmy profile is a partially inherited individual characteristic.

In conclusion many findings showed that exceptional longevity runs in families [1-4], but to date genetic studies on longevity and healthy aging have shown that centenarians carry the same numbers of GWAS-identified common disease variants of ordinary people. In this context rare and private variants seem to have a relevant role in buffering the presence of other variants [48]. Thus we reasoned that the long-term heteroplasmy inheritance, that we demonstrate in the present study, is a potential source of rare variants that could play a role in determining healthy aging and longevity. The issue is to demonstrate the effect of these patterns on longevity, but this limitation is also valid for all the rare variants that can be described in whole genome.

Other studies had already shown that the description of heteroplasmy is complicated by the characteristics of the blood itself such as the origin of the cells, the rapid turnover and the high number of different cell types [27]. We can speculate that at the somatic level rare heteroplasmic mutations can expand or be lost, through segregation events. Thus in a given anatomical region such as muscle or brain, heteroplasmies expansion occurs and remains localized in the same physical area [37]. Similarly this may occur during hematopoietic cell expansion in the bone marrow but the local heteroplasmy profiles are diluted when the new cells are released in the bloodstream. This implies that the heteroplasmic variants that we describe at low frequencies in blood are compatible with the high heteroplasmy level detected in other tissues (such as skeletal muscle and brain). It is likely that low level heteroplasmies have the potential to increase in frequency in critical tissues. Further studies considering different tissues of the same individual are needed to experimentally test this last consideration and to differentiate between acquired or inherited heteroplasmic mutations [24]. Indeed the exact mechanism of inheritance as well as the origin of these heteroplasmic events are not completely understood, and the forces that shape heteroplasmies are under study [29, 49, 50]. Evidence from mice and humans suggests that nonsynonymous protein-coding heteroplasmies are subject to negative selection during growth and aging [31, 50]. A further critical issue concerns the minimum percentage of detectable mutations compatible with the size of the bottleneck that can be observed in blood and the exact timing of these mutations. Many studies have attempted to estimate the size of the bottleneck [29, 51], but more data are necessary to answer, at least on a theoretical level, this question.

Finally these findings add significantly to our knowledge of mechanisms of heteroplasmy transmission and association with longevity, and indicate that heteroplasmic events are an interesting candidate for further genetic studies on familial longevity. Our results are compatible with recent evidence that shows that the transmission of low levels of mtDNA heteroplasmy seems to influence offspring aging [37, 52], and by focussing on long-lived individuals and their offspring, adds a new perspective on the contribution of low level heteroplasmies to the genetics of longevity.

## METHODS

### Sample collection

A total of 90 Italians were sequenced, including 31 female centenarians (average age 101.6 ± 2.8), 31 female offspring of these centenarians (average age 69.9 ± 6.6) and 28 female offspring whose parents both died before the average life expectancy (average age 70.7 ± 4.8). The samples here analyzed were selected according to a stringent demographic strategy: centenarians and offspring were recruited with the constraint that also the other parent was long lived (born in the same birth cohort 1900-1908 and died at an age > 77 years). Offspring of non-long lived parents were matched with centenarian offspring, with both parents born between 1900 and 1908 but dying before the average life expectancy calculated at 15 years of age (67 years if male and 72 years if female) by the Italian mortality tables (see http://www.mortality.org/ - Max Planck Institute for Demography, Rostock, Germany). Both groups were comparable with respect to age but very different if we considered the probability of becoming long lived [34]. Indeed recent studies suggest that the offspring of centenarians have a significant survival advantage, a higher probability to become long-lived and a lower risk for age-related diseases (such as cardiovascular diseases) compared to control subjects [4, 5, 45, 53]. Individuals with cancer at the time of the interview or treated with immunosuppressive or anticoagulant therapies were excluded from this study.

Genomic DNA was extracted from whole blood using the Wizard genomic DNA purification kit (PROMEGA, Madison WI, USA) according to the manufacturer's standard protocols.

### Ethics statement

The study protocol was approved by the Ethical Committee of the Sant'Orsola - Malpighi University Hospital (Bologna, Italy). After obtaining written informed consent, a standard structured questionnaire was administered to collect information regarding health status. Past and current diseases were checked against the medical documentation.

### DNA amplification and sequencing

DNA was first quantified by NanoDrop (NanoDrop Products Thermo Scientific Wilmington, DE) and an 853 bp segment of the mtDNA control region (from position 15978 to 261, including HV1 and part of HV2) was amplified. We focus on this region because heteroplasmies occur preferentially at positions with high mutation rates, like the mtDNA control region, and also because this region was already analyzed in previous studies on longevity. Primers were selected after in silico PCR to avoid amplification of unspecific nuclear DNA (L15997, CACCATTAGCACCCAAAGCT and H242, GCTGTGCAGACATTCAATTG). Amplified fragments were then sheared using the Bioruptor UCD-200 (Diagenode) to approximately 200 – 800 bps. Libraries were prepared following a previously published protocol [54]. Two different indexes were attached to individual samples by performing a PCR amplification using the Herculase Mastermix (NEB). Samples were pooled in equimolar ratios after indexing and subsequently sequenced in one lane of the Illumina GAIIx (Solexa) sequencer, with a paired-end run of 76+7 cycles, which yielded ~45 million paired-end reads.

### Assembly and data analysis

Base quality scores were recalibrated by the IBIS software using spiked-in PhiX sequencing data as the training dataset [55]. The adaptor sequence was trimmed and paired-end reads were merged if they were completely overlapped. Reads were mapped to the revised Cambridge reference sequence (GenBank ID:NC_012920, the starting point was changed to 15978 on the original sequence) using BWA [56]; reads with mapping quality score lower than 20 and bases with quality score lower than 20 were excluded from the analysis, resulting in a final average coverage of 49334-fold per position in the target region.

Samples that showed low coverage (mean coverage <10000) were excluded from the analysis of heteroplasmy. Mean coverage distribution is reported in Figure 4S. Initially 31 CENT, 31 CO and 28 ONLL were sequenced but 1 CENT and 1 unrelated CO did not pass the quality control (Figure 4S) and consequently for the centenarians-offspring paired analysis there were 29 CENT-CO pairs. Analyses were performed using R version 3.0.1. We called heteroplasmic sites according to minor allele frequency even if some heteroplasmic positions were then filtered out after double strand validation, as described previously [36].

The heteroplasmy detection threshold was set to 0.2%; the sensitivity and specificity of detected heteroplasmies were determined from 12 samples (one sample was discarded after quality filters) whose levels of heteroplasmy were measured using 3 different PCR products. The concordance was used as a parameter of reliability in the definition of heteroplasmic sites. For this analysis we create a confusion matrix using the *caret packages* and in this calculation a site was defined as “true heteroplasmic” if heteroplasmy was supported by at least 2 of the 3 observations [57-59]. The overview of heteroplasmy pattern (Figure 1) was perfomed using the *lattice* graphics package [60]. The mutation rate was estimated for each site from Phylotree [61]. Unsupervised hierarchical clustering was performed on the basis of the *complete linkage* method, using the function heatmap.2 in the *gplots package*.

## SUPPLEMENTARY FIGURES AND TABLES


