# Supplementary material for: Transmission from centenarians to their offspring of mtDNA heteroplasmy revealed by ultra-deep sequencing
Source: Aging (Albany NY). 2014 May 13;6(6):454–67. doi: 10.18632/aging.100661 (PMC4100808; doi:10.18632/aging.100661)
Supplement: Supplementary file 1 [file aging-06-454-s001.pdf]

SUPPORTING INFORMATION

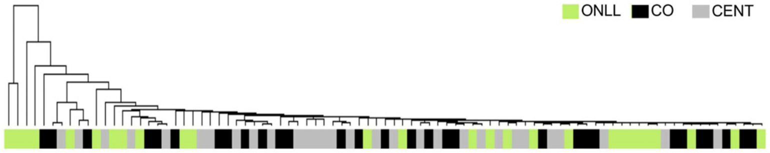

Figure 1S. Unsupervised hierarchical clustering using heteroplasmy levels. CENT: gray bar; CO: black bar; ONLL: green bar.

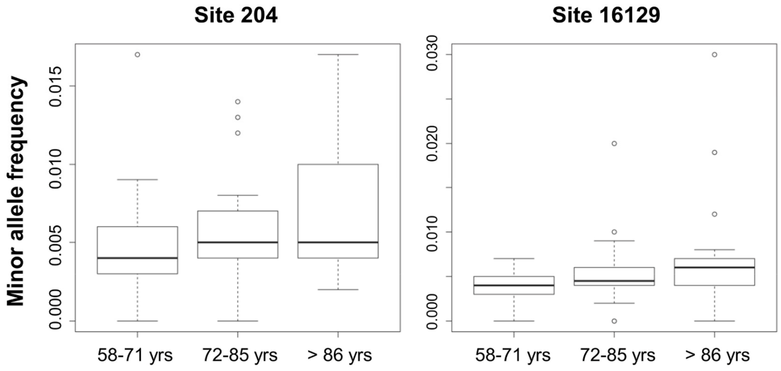

Figure 2S. Boxplots of minor allele frequencies across 3 different age classes. Age < 72 yrs; N=31, age range: 72-86 yrs; N=26 and age > 86 yrs; N=29 respectively for two heteroplasmic sites

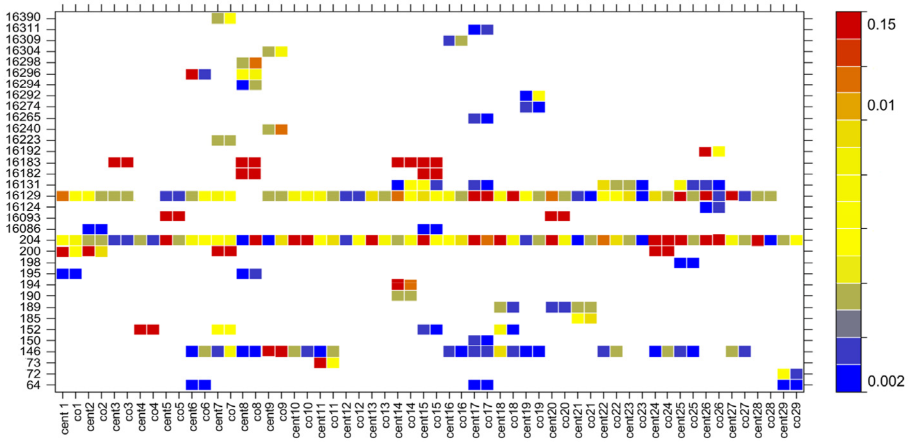

Figure 3S. Heatplot of minor allele frequencies, colored according to the scale bar, for each mother-offspring pair. Rows are positions and columns are individuals.
